# Pharmacokinetics of Human Recombinant Anti-Botulinum Toxin Antibodies in Rats

**DOI:** 10.3390/toxins11060345

**Published:** 2019-06-17

**Authors:** Yero Espinoza, David Wong, Ago Ahene, Kenneth Der, Zachary Martinez, John Pham, Ronald R. Cobb, Shauna Farr-Jones, James. D. Marks, Milan T. Tomic

**Affiliations:** 1 Ology Bioservices Inc., 630 Bancroft Way, Suite D, Berkeley, CA 94710, USA; yero.espinoza@ologybio.com (Y.E.); david.wong@ologybio.com (D.W.); zach.martinez@ologybio.com (Z.M.); 2 Fiveprime Therapeutics Inc., 111 Oyster Point Blvd, South San Francisco, CA 94080, USA; ago.ahene@fiveprime.com; 3 Portola Pharmaceuticals Inc., 270 E Grand Ave, South San Francisco, CA 94080, USA; kder@portola.com; 4 OncoMed Inc., 800 Chesapeake Dr, Redwood City, CA 94063, USA; Jtpham2015@yahoo.com; 5 Ology Bioservices Inc., 13200 NW Nano Ct, Alachua, FL 32615, USA; ron.cobb@ologybio.com; 6 Department of Anesthesia and Perioperative Care, University of California, San Francisco, 1001 Potrero Ave, San Francisco, CA 94110, USA; shauna.farr-jones@ucsf.edu (S.F.-J.); jim.marks@ucsf.edu (J.D.M.)

**Keywords:** botulinum neurotoxin, pharmacokinetics, recombinant antibody, rat, oligoclonal antibody, anti-botulinum neurotoxin antibody

## Abstract

Botulinum neurotoxins (BoNT) are potential biothreat agents due to their high lethality, potency, and ease of distribution, thus the development of antitoxins is a high priority to the US government. This study examined pre-clinical pharmacokinetic studies in rats of four oligoclonal anti-BoNT mAb-based therapeutics (NTM-1631, NTM-1632, NTM-1633, NTM-1634) for five BoNT serotypes (A, B, E, C, and D). NTM-1631, NTM-1632, and NTM-1633 each consist of three IgG1 mAbs, each with a distinct human or humanized variable region which bind to distinct epitopes on BoNT serotype A, B, or E respectively. NTM-1634 consists of four human immunoglobulin G1 (IgG1) mAbs binding BoNT C/D mosaic toxins. The mechanism of these antitoxins requires that three antibodies simultaneously bind toxin to achieve rapid clearance. Rats (total 378) displayed no adverse clinical signs attributed to antibody treatment from any of the antitoxins. Pharmacokinetic evaluation demonstrated that the individual mAbs are slowly eliminated, exhibiting dose-dependent exposure and long elimination half-lives ranging from 6.5 days to 10 days. There were no consistent differences observed between males and females or among the individual antibodies in each formulation in half-life. Anti-drug antibodies (ADA) were observed, as expected for human antibodies administered to rats. The results presented were used to support the clinical investigation of antibody-based botulism antitoxins.

## 1. Introduction

Botulinum toxin (BoNT) is the most potent toxin known, with a long duration of action. BoNT intoxication results in an acute, descending, symmetric flaccid paralysis caused by neurotoxin-mediated blockade of the presynaptic acetylcholine release. Treatment requires prolonged hospitalization [1,2,3,4]. Human botulism is caused by botulinum neurotoxin (BoNT) serotypes A, B, E, F [5,6,7,8,9], C, D, [10,11,12] and G [13]. 

Due to their lethality and ease of distribution, BoNTs are classified as Category A agents by the US Centers for Disease Control and Prevention (CDC) [14]. They are also Tier 1 agents as defined by the Federal Select Agent Program and are among the six agents with the highest risk for potential use as bioweapons. For example, both Iraq [15] and the former Soviet Union produced BoNT for use as weapons [16]. As a result of these threats, there is an urgent need for safe and effective therapies that could be stockpiled, as well as sensitive and rapid diagnostic tests. 

The current standard of care for botulism is polyclonal antitoxin from either human or equine immunization [17,18,19,20]. Human botulism immunoglobulin, BabyBIG®, is produced by plasmapheresis of lab workers who were immunized with an investigational toxoid vaccine, and is costly [20]. Furthermore, an investigational pentavalent vaccine was recently withdrawn from clinical use due to potency concerns, and currently, no vaccine is available, thus limiting future production of BabyBIG. Equine antitoxin (BAT®) [21], comprised of immunoglobulin from serum of immunized horses, has been proteolyzed to remove the Fc (fragment crystallizable) portion, resulting in over 90% Fab (antigen binding fragment) and F(ab’)_2_. BAT, developed by Cangene Corp. (Winnipeg, Manitoba, Canada) has been used for botulism treatment, but it has a short half-life and can induce acute allergic responses and serum sickness, as well as induce antibodies against the equine antitoxin components [19]. BAT is also very expensive to produce—200,000 doses were manufactured under a $363 million US government contract. For all these reasons, the US National Institute of Allergy and Infectious Diseases (NIAID) has funded discovery and development of next generation antitoxins for the treatment of botulism comprised of recombinant human monoclonal antibodies (mAbs) [17,22,23,24,25]. Recombinant human monoclonal antibodies (mAbs) would provide a renewable, and safe antitoxin that could be easily administered in mass casualty situations and sporadic cases.

Previous work found that no single anti-BoNT mAb effectively neutralizes the toxin [17,26]. A combination of three mAbs is required to effectively neutralize BoNT [17]. The oligoclonal antitoxins NTM-1631, NTM-1632, NTM-1634, NTM-1633 were developed to treat and prevent botulism due to BoNT/A, BoNT/B, BoNT/C/D and BoNT/E, respectively. NTM-1631, NTM-1632, NTM-1633 are equimolar mixtures of three IgG1 mAbs. Each mAb targets different and non-overlapping epitopes of BoNT. NTM-1634 is an equimolar mixture of four human mAbs. The antibodies bind serotypes C or D or both (unpublished).

Since the mechanism of action of these antitoxins requires the presence of all three antibodies in the mixture [17], determining the pharmacokinetics (PK) of the individual antibodies is important for determining the window of protection. The requirement for three antibodies to clear toxin means that the most rapidly cleared antibody determines protection. While the antibody mixtures are all constructed using identical framework regions, different variable region sequences were expected to affect clearance rates.

To determine the PK of each antibody, antibody-specific assays were developed. Quantitation of individual mAbs within an oligoclonal mixture targeting the same antigen presents significant technical challenges. To support pre-clinical and clinical PK studies, manufacturing and stability studies of the drug product, methods for quantitation of each mAb comprising the mixture are needed. Domain-based assays for the individual antibodies of oligoclonal mixtures of antibodies were previously developed that bind BoNT serotypes A [27], B and E [28], and C/D (unpublished). Protein domains with selected mutations were designed so that they bound only one antibody in the mixture. 

Rat PK parameters for the four antitoxins reported here were used to support clinical studies with the goal of developing these drugs as a botulism treatment and post-exposure prophylaxis.

## 2. Results

### 2.1. Safety

No adverse clinical signs attributed to treatment were observed in any dose groups after dose administration of any of the drug products. Sporadic clinical observations were observed in the groups that were administered the antibody mixtures. The clinical observations that were observed included chromodacryorrhea, exophthalmia, microphthalmia, red discharges in the noses or genitalia, alopecia, and soft stool. These effects were determined to be independent of dose by the attending veterinarian and most likely the result of the stress due to the dose administration procedures. This is because the observations occurred in 1 or 2 rats in any dose group and the observations were transient and did not persist, or they were common observations associated with restraint for blood collection.

### 2.2. Pharmacokinetic Analyses NTM-1631

**Figure 1:**
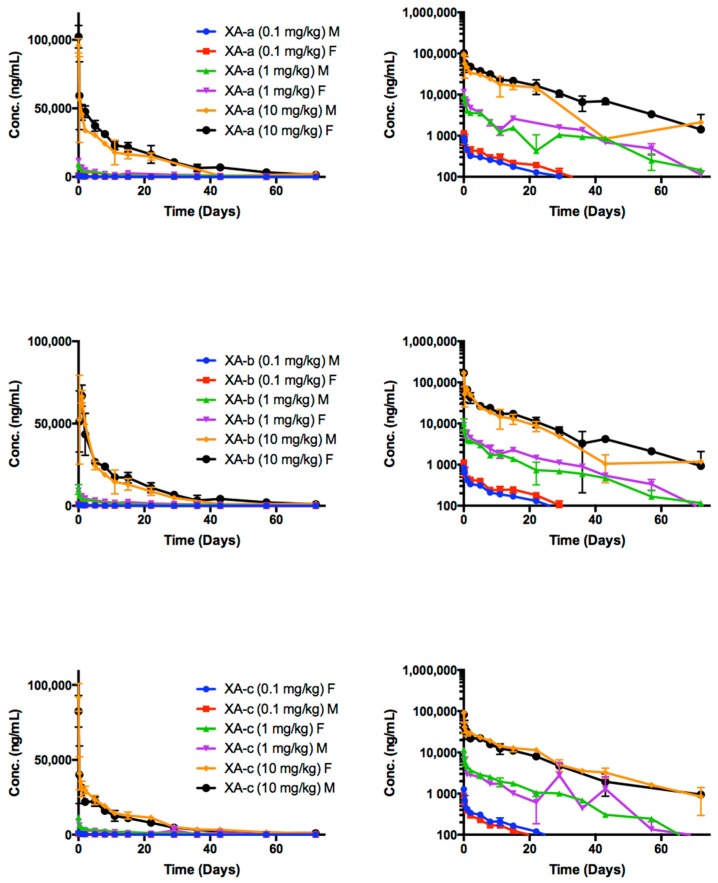
The time course of rat serum mAb concentrations after single intravenous (IV) dose administration of NTM-1631. NTM-1631 component mAbs are denoted XA-a, XA-b, XA-c. The animals were dosed with a single IV bolus of 0.1, 1.0 and 10 mg/kg of drug product. Log concentration plots are shown on the right. The data presented are from rats negative for immunogenicity against drug only (no anti-drug antibodies (ADA), See Table S1). The points represent the mean ± SE of n = 3 or more.

Time course of NTM-1631 component mAbs in rat sera are shown in Figure 1. NTM-1631 component mAbs are denoted XA-a, XA-b, XA-c. The PK parameters were calculated for anti-drug antibodies (ADA)-negative animals in the study (Table 1). The T_1/2_ ranged from: XA-a, 14.61 days (10 mg/kg, females) to 17.23 days (0.1 mg/kg, males); XA-b, 13.40 days (1 mg/kg, females) to 15.82 days (0.1 mg/kg, males); XA-c, 12.36 days (1 mg/kg, females) to 16.17 days (0.1 mg/kg, males). There were no consistent differences observed in PK between males and females or amongst the three antibodies.

**Figure 2:**
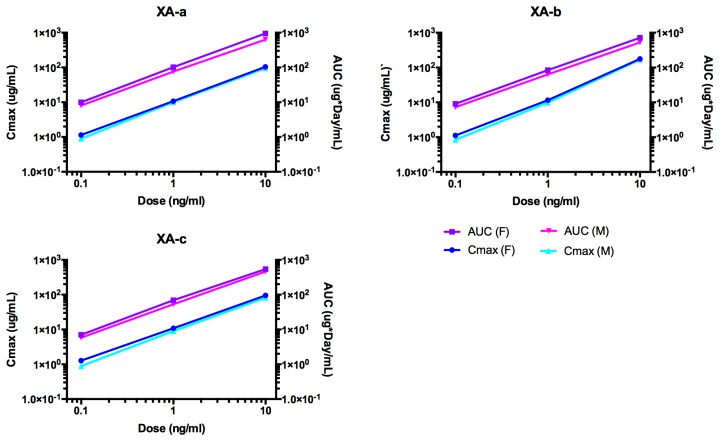
Dose-dependent C_max_ (maximum observed mean concentration) and AUC (area under the curve) of individual monoclonal antibodies (mAbs) for male and female rats after single IV administration of NTM-1631. The data presented are from rats with non-ADA only, see Table S1.

Indicators of exposure, maximum concentration (C_max_) and area under the curve (AUC) values, increased approximately linearly with the dose for XA-a, XA-b, and XA-c as shown in Figure 2. Only the observed C_max_ for XA-b in the 10 mg/kg dose group was higher than the C_max_ for XA-a and XA-c. This resulted in a greater than linear increase in this parameter for XA-b. The AUC_last_ for all three antibodies increased in a dose-dependent manner. Based on the AUC_last_, exposure to each of the mAbs was higher in females than males. In both males and females, exposure was highest for XA-a, followed by XA-b and then XA-c based on AUC data. The AUC_∞_ values were essentially the same as the AUC_last_ values, as would be expected since serum samples were collected through 72 days after dose administration or approximately 5 half-lives. The volume of distribution of the central compartment (Vc) values were similar for all three mAbs and all dose groups. The values varied from 69.58 mL/kg (XA-a, 1 mg/kg, females) to 125.88 mL/kg (XA-c, 0.1 mg/kg, males) suggesting distribution to the vascular space. The result for Cl tended to be slightly higher in male than female rats, for each mAb and dose level, as expected based on the slightly higher exposure observed for females relative to males. 

### 2.3. ADA of NTM-1631

An ADA response depends primarily on immunogenicity of the drug, dose frequency, and route. Human proteins are expected to be immunogenic to rats and did produce an ADA response in 30 of 90 NTM-1631-treated rats. At the lowest dose group (0.1 mg/kg), ADA were detected in only one animal. In the mid-dose group (1 mg/kg), 47% of the animals (8/15 males and 6/15 females) were ADA-positive and 50% in the high dose group (10 mg/kg) were ADA-positive (10/15 males and 5/15 females).

Antibodies against XA-a, XA-b, and XA-c in the serum were evaluated for the last time-point for each animal at the terminal blood collection (3 rats/sex/group at days 29, 36, 43, 57, and 71). In the low dose group, only one rat at a single timepoint elicited an antibody response to NTM-1631 (Table S1). The data from ADA-positive rats were not used in calculations of PK parameters. 

### 2.4. Pharmacokinetic Analyses NTM-1632

**Figure 3:**
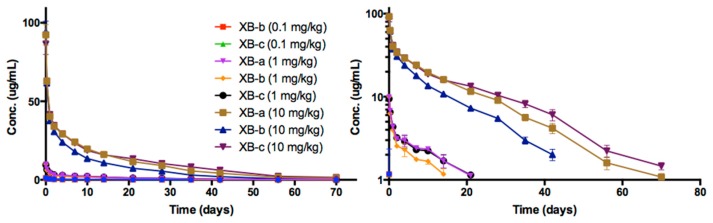
The time course of rat serum mAb concentrations after single IV dose administration of NTM-1632. NTM-1632 component mAbs are denoted XB-a, XB-b, XB-c. Animals were dosed with a single IV bolus of 0.1, 1.0 and 10 mg/kg of drug product. Log concentration plots are shown on the right. The data presented are from rats negative for immunogenicity against drug only (no ADA, See Table S1). The points represent the mean ± SE of n = 3 or more.

**Figure 4:**
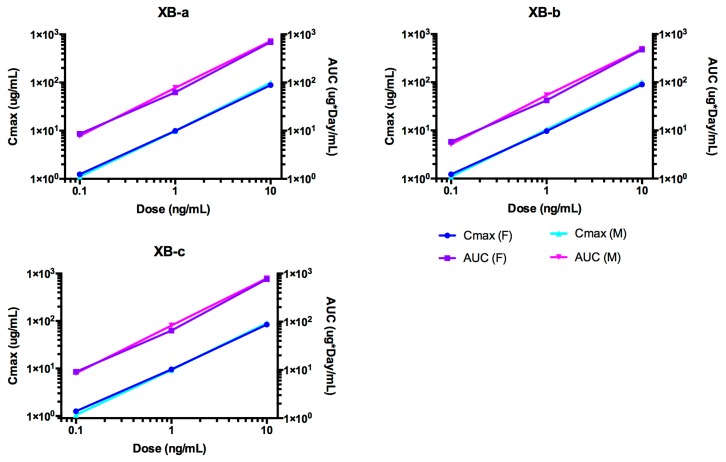
Dose-dependent C_max_ and AUC for rats after single IV administration of NTM-1632. The data presented are from rats with no ADA.

The time course of NTM-1632 antibodies in rat sera are shown in Figure 3. NTM-1632 component mAbs are denoted XB-a, XB-b, XB-c. The PK parameters were calculated for ADA-negative animals in the study (Table 2). Indicators of exposure, C_max_ and AUC values, increased approximately linearly with the dose for all three antibodies as shown in Figure 4. The C_max_ values were similar among dose levels for each of the three mAbs in NTM-1632. No sex differences were observed in the concentration at various times, thus data from male and female rats were pooled. Noncompartmental analyses demonstrated that all three mAbs displayed similar T_1/2_ ranging from: XB-a, 13.6 days (10 mg/kg, females) to 17.9 days (0.1 mg/kg, males); XB-b, 9.15 days (0.1 mg/kg, males) to 20.3 days (0.1 mg/kg, females); and XB-c 13.3 days (10 mg/kg, females) to 19.6 days (0.1 mg/kg, males). Vc were similar for all three antibodies at all dose groups and tended to be lower in males than in females for all three mAbs, although the differences were small, suggesting distribution to the vascular space. The Cl values were also lower in males, which is consistent with the higher AUC observed in male rats. Note that in Figure 3, data points for the 0.1 mg/kg dose are overlapping in linear and logarithmic plots.

### 2.5. ADA of NTM-1632

Only five out of the 90 rats were ADA-positive (Table S1). At the lowest dose group (0.1 mg/kg), only one animal was detected to be ADA-positive. In the middle dose group (1 mg/kg) and high dose group (10 mg/kg), two animals were ADA-positive from each group. By day 35, most of the ADA-positive animals had serum concentrations of the mAbs that were at or near the lower limit of quantification (LLOQ) of the assay. The data from the ADA-positive animals from this study were not used in further calculations of the PK parameters.

### 2.6. Pharmacokinetic Analyses NTM-1633

**Figure 5:**
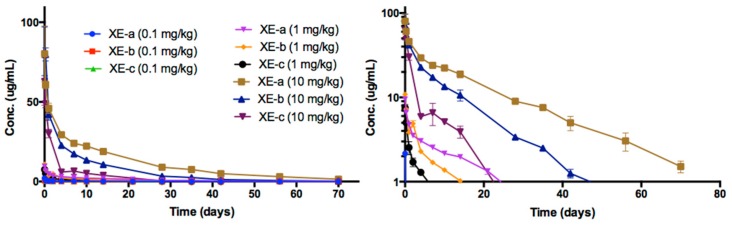
The time course of rat serum mAb concentrations after a single IV dose administration of NTM-1633. Animals were dosed with a single IV bolus of 0.1, 1.0 and 10 mg/kg of drug product. Log concentration plots are shown on the right. The data presented are from rats negative for immunogenicity against drug only (no ADA, See Table S1). Points represent the mean ± SE of n = 3 or more.

**Figure 6:**
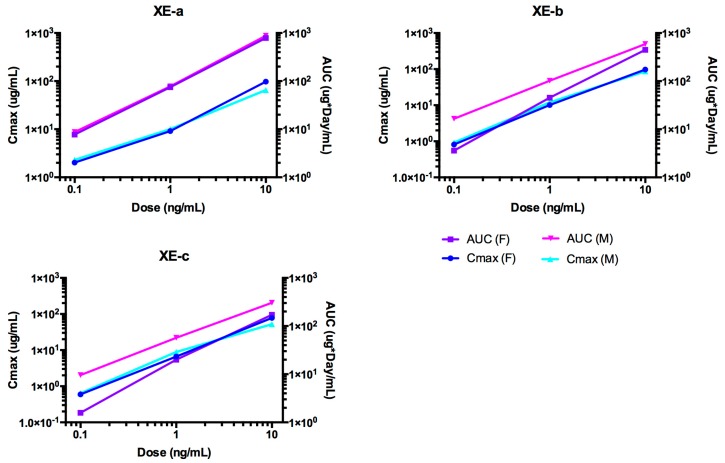
Dose-dependent C_max_ and AUC for rats after single IV administration of NTM-1633. The data presented are from rats with no ADA.

The time course of NTM-1633 antibodies in rat sera are shown in Figure 5. NTM-1633 component mAbs are denoted XE-a, XE-b, XE-c. The PK parameters were calculated for ADA-negative animals in the study (Table 3). The indicators of exposure, C_max_ and AUC values, increased approximately linearly with dose for all three antibodies as shown in Figure 6. The mean NTM-1633 antibody concentrations showed a biexponential decline in the time-concentration curve at each dose level (0.1, 1.0, and 10 mg/kg) and for each antibody (XE-a, XE-b, and XE-c). Noncompartmental analysis showed similar elimination half-lives between the antibodies XE-a and XE-b with overall mean values of 17.3 and 17.5 days, respectively, and a shorter overall mean half-life of 13.5 days for XE-c. Clearance varied among the antibodies with overall mean values of 4.21, 7.58, and 17.8 mL/day/kg for XE-a, XE-b, and XE-c, respectively. Exposure-based on AUC∞ between antibodies was consistently greater for XE-a, followed by XE-b and then XE-c at each dose level, which is consistent with the trend that higher clearance lowers AUC. Overall, C_max_ also showed linearity among all the dose levels within each antibody, except for the 0.1 mg/kg XE-a C_max_, which showed greater than expected maximum concentration relative to its 1.0 and 10 mg/kg dose. Vc was similar for all three antibodies with an overall average of 35.1, 38.1, and 52.1 mL/kg for XE-a, XE-b, and XE-c, respectively. No sex differences were observed in the concentration-time plots of each antibody. Therefore, because data points for male and female rats would overlap, data from male and female was pooled for these time plots. In Figure 5, data points for the lowest dose, 0.1 mg/kg, are closely overlapping.

### 2.7. ADA of NTM-1633

Twenty-six animals out of 90 rats were ADA-positive: seven (23.3%) in the 1 mg/kg and 19 (63.3%) in the 10 mg/kg dose groups, with no ADA in the 0.1 mg/kg dose group (Table S1). Clearance from all ADA-positive animals for each antibody increased relative to the animals testing ADA-negative. By Day 42, most ADA-positive animals had mAb concentrations at or near the LLOQ of the assay.

### 2.8. Pharmacokinetic Analyses NTM-1634

**Figure 7:**
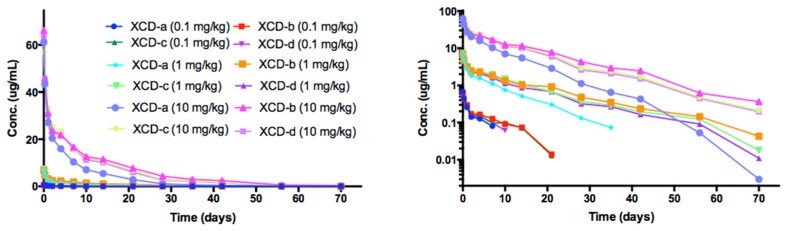
The time course of rat serum mAb concentrations after single IV dose administration of NTM-1634. The animals were dosed with a single IV bolus of 0.1, 1.0 and 10 mg/kg of drug product. Log concentration plots are shown on the right. The data presented are from rats negative for immunogenicity against drug only (no ADA, See Table S1). The points represent the mean ± SE of n = 3 or more.

**Figure 8:**
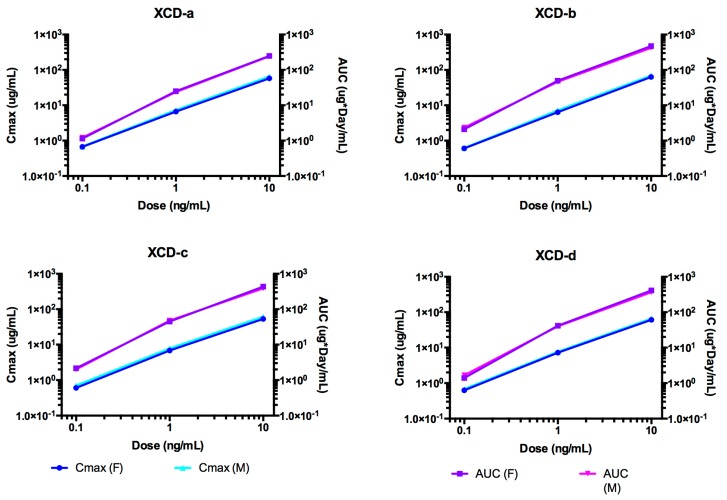
Dose-dependent C_max_ and AUC for rats after single IV administration of NTM-1634. The data presented are from rats with no ADA.

The time course of NTM-1634 antibodies in rat sera are shown in Figure 7. NTM-1634 component mAbs are denoted by XCD-a, XCD -b, XCD -c, XCD-d. The PK parameters were calculated for ADA-negative animals in the study (Table 4). Indicators of exposure, C_max_ and AUC values, increased approximately linearly with the dose for all three antibodies as shown in Figure 8. The T_1/2_ was approximately 7 to 10 days for the lowest dose for four NTM-1634 antibodies. The 1 and 10 mg/kg dose groups had half-lives of 9 to 10 days for these same antibodies. All four antibodies had a similar response for all dose groups as shown in Figure 1. XCD-a antibody showed a more rapid clearance profile than the other antibodies at all three doses. The other three antibodies had a similar response for all three doses.

The T_1/2_ for the XCD-a was approximately 30% less than the other three antibodies across the three dose groups and showed a more rapid clearance profile for all dose groups. Observed antibody clearance profiles are typical for rats injected with human antibodies. Vc values were similar for all four antibodies at all dose groups. 

### 2.9. ADA of NTM-1634

Nineteen out of the 108 rats were ADA-positive (Table S1). At the lowest dose group (0.1 mg/kg), three animals were confirmed to be ADA-positive. In the middle dose group (1 mg/kg) and high dose group (10 mg/kg), 14 animals were ADA-positive from each group. By day 35, most of the ADA-positive animals had serum concentrations of the mAbs that were at or near the LLOQ of the assay. The presence of ADA in some of the animals did not significantly affect the PK as the difference between the PK parameters with and without ADA animals was not significantly different. In Figure 7, data points for the lowest does, 0.1 mg/kg are overlapping.

### 2.10. Relationship of Half-Life and Clearance to pI

Each of the 13 IgG1 antibodies studied here has the same constant region, with different variable regions. It was therefore considered whether pI was correlated with clearance and T_1/2_ as previously reported for IgG4 antibodies [29]. It was found that predicted pI was uncorrelated with half-life (r squared = 0.0036, data not shown).

## 3. Discussion

Botulinum neurotoxins are among the most toxic known compounds [30], yet there is no preventive agent for botulism that would be logistically feasible should large numbers of people be exposed to BoNT via either food or intentional exposure by those of ill intent. Next generation recombinant human antitoxins for BoNTs are in clinical development, funded by NIAID. These recombinant antibody-based drugs address the many drawbacks of equine antitoxin (BAT) [21], namely, adverse events due to immunogenicity of non-human antibodies, lack of lot-to-lot reproducibility, high cost of goods, and a short half-life. These recombinant antibodies could also replace the human-derived immune globulin antitoxin (BabyBIG) [20], for which future production is in jeopardy due to lack of an approved human vaccine [31]. 

Any prophylactic or therapeutic treatment for botulism due to food poisoning should have an extended half-life in humans because colonization of the gut by clostridial species would produce toxins continuously, and a long half-life would allow prophylactic use of antibodies. Furthermore, an extended half-life allows for prophylactic use of antitoxins, should potential exposure to toxins be anticipated, such as in the case of emergency responders or military personnel. The short half-life of BAT precludes its use for prevention of botulism, since protective serum levels of antibody are short lived, on the order of hours to 0.33 to 2 days [21]. This study provides the PK parameters of NTM-1631, NTM-1632, NTM-1633 and NTM-1634 administered IV in rats. 

With respect to safety in rats, administration of these human recombinant antibody drug products by slow bolus IV injection was well tolerated in Sprague Dawley rats at levels up to 10 mg/kg/dose, being the highest dose for these studies. Rats showed no significant changes in clinical findings, serum chemistry, hematology, urinalysis, or histopathology at a dose of 50 mg/kg delivered IV for any of the four antitoxins. The escalating doses of each were well tolerated with no adverse effects that were drug specific. The safety of these antitoxins in humans so far, reflects that observed in rats, namely that no significant treatment associated adverse effects were observed [24], and unpublished results.

As these are human antibodies, ADA were expected and observed in rats. ADA-positive animals were more prevalent in the mid and high dose groups compared to the low dose group. The drugs were cleared from serum more rapidly with a shorter T_1/2_ and lower volume of distribution. 

Previous studies have shown that the effectiveness of mAbs in neutralizing BoNTs is dependent on the presence of three antibodies against each BoNT [17]. The duration of the effectiveness of the combination may be determined when the level of a single antibody drops below the effective dose levels most rapidly. In human studies, the humanized mouse mAb was cleared most rapidly at the lowest dose, but all three mAbs comprising NTM-1631 were detected for a minimum of 4 weeks after infusion [24]. In the present study, all mAbs in each of the four drug products were detected up to 72 days after infusion. Similarly, PK results in a guinea pig model demonstrated that after IV injection, mAb concentrations were able to show protection in a mouse neutralization assay up to 14 days post injection [32].

The PK behavior of the individual antibody components of the four drug products (NTM-1631, NTM-1632, NTM-1633, NTM-6134) show a biphasic decline similar to other monoclonal antibodies. Each antibody exhibited dose-dependent exposure and long elimination half-lives of at least 7 days after IV administration of 0.1, 1, and 10 mg/kg in Sprague Dawley rats. Rat half-life was largely predictive of the rank order of half-life observed in humans. For NTM-1631, the rank order in rats and humans was XA-c > XA-b > XA-b [24] and for NTM-1634, the rank order in both rats and humans was XCD-b > XCD-c > XCD-d > XCD-a (Snow et al., manuscript submitted). In rats, the most rapidly cleared mAbs are XA-c (T_1/2_ 12.7 ± 2.8 days), XB-b (T_1/2_ 14.9 ± 2.3 days), XCD-a (T_1/2_ 6.5 ± 2 days), and XE-c (T_1/2_ 13.5 ± 4.5 days).

Previous studies have demonstrated that the oligoclonal recombinant antibody antitoxins reduced the mortality in mice when administered prior to BoNT challenge [17,33,34,35,36], and post-challenge in mice [37]. Finally, NTM-1631 and NTM-1632 have been shown to protect guinea pigs from challenge with inhaled BoNT when the antibodies were administered prior to challenge [32]. 

## 4. Conclusions

Rat PK studies of the four antitoxins (NTM-1631, NTM-1632, NTM-1633, NTM-6134) were used to support investigational new drug (IND) applications to the FDA and to guide design of first in human studies. 

## 5. Materials and Methods 

All studies were performed in accordance with the U.S. FDA “Good Laboratory Practice for Nonclinical Laboratory Studies” (GLP) as described in 21 CFR Part 58. All procedures were approved by the Animal Ethics Committees of SRI, protocol B311-08 (single dose PK protocol approved 15 July 2008) for NTM-1631; and Charles River Laboratory single-dose PK protocol 20021165 (approved 22 April 2013), multi-dose toxicology protocol 20021158 (approved 23 October 2013), for NTM-1632; single-dose PK protocol 20022284 (approved 15 April 2013), multi-dose toxicology study 20022285 (approved 23 October 2013) for NTM-1633; and dose finding toxicology protocol 20066727 (approved 26 May 2015), single dose PK study 20074281 (approved 26 May 2015), multi-dose toxicology 20081234 (approved 2 September 2015) for NTM-1634.

### 5.1. Preparation of Test Articles 

All four antitoxins are composed of equimolar mixtures of three or four monoclonal antibodies (mAbs). NTM-1631 is composed of two human and one humanized mAbs (XA-a, XA-b, XA-c). NTM-1632 is composed of three human mAbs (XB-a, XB-b, XB-c), NTM-1633 composed of three human mAbs (XE-a, XE-b, XE-c) and NTM-1634 composed of four human mAbs (mAb XCD-a, XCD-b, XCD-c, XCD-d). Concentration of mAbs in each product was 5 mg/mL for NTM-1631 and NTM-1632, 5.1 mg/mL for NTM-1633 and NTM-1634. All test articles were stored at 4 °C until use. Test articles were prepared by dilution using 10 mM sodium succinate/succinic acid and 142 mM L-arginine-HCl and 0.005% polysorbate 80, pH 6.0, and were diluted and mixed for 20 min and then stored on wet ice prior to dose administration. Diluted test articles were prepared fresh on the day of dosing and brought to room temperature prior to administration to the animals. Formulation accuracy was verified by ELISA.

### 5.2. Animal Care

The study included male and female Sprague Dawley rats (Harlan Laboratories, Indianapolis, IN) for the NTM-1631 studies, and (Charles River Laboratories, Hollister, CA, USA) for the NTM-1632, NTM-1633 and NTM-1634 studies. Rats were divided into groups each with 15 male and 15 female rats (or 18 each male and female for the NTM-1634 study). The total number of rats for the entire study was 378 (90 each for NTM -1631, NTM-1632, NTM-1633 and 108 for NTM-1634). Male rats were 7.5 to 11 weeks of age and weighed 221–350 g. Female rats were 8.5 to 11 weeks of age and weighed 171–275 g. The dose administration schedule and study endpoints were clinical observations, body weights, and PK analysis by electrochemiluminescence (ECL).

General procedures for animal care and housing were in accordance with the National Research Council (NRC) Guide for the Care and Use of Laboratory Animals (1996) and the Animal Welfare Standards Incorporated in 9 CFR Part 3, 1991, and conformed to the testing facility SOPs. Animals were received and quarantined for 3-5 days during which time they were examined by a veterinarian, who assessed their health for suitability for use on-study. Animals were housed three or fewer per cage during quarantine and during the study in suspended polycarbonate cages (with bedding) on stainless steel racks. Rats were provided water and certified diet *ad libitum*. 

All animals were euthanized with an overdose of sodium pentobarbital administered intraperitoneally after the last blood collection. The lack of heart beat was used to establish death. This method is acceptable according to the American Veterinary Medical Association Guidelines for Euthanasia [38].

### 5.3. Dose Administration

The animals were dosed by a single IV injection of 0.1, 1 or 10 mg/kg/dose into the lateral tail vein over ~45–60 s once on Day 1. The dosing volume was 2 mL/kg (10 mg/kg) based on the most recent body weights. The animals were temporarily restrained for dose administration and were not sedated.

### 5.4. Clinical Observations

Observations for mortality and moribundity were performed at least daily. Pre-test cage-side observations for general health were conducted on all animals twice daily. Cage-side clinical observations for morbidity were conducted at least twice on days of dose administration (once before dose administration and at a target time of 1-h following dose administration) and at least once daily on the days without dose administration.

### 5.5. Blood Sample Collection

Blood samples were collected and processed for serum drug levels at pre-dose, 5 min, 6, 24, and 48 h post-dose and once on days 5, 8, 11, 15, 22, 29, 36, 43, 57 and 71 or 72. Three rats per sex were evaluated for each time-point. Blood was collected from the retro-orbital sinus of rats under 60:40% CO_2_:O_2_ anesthesia or from the jugular (preferred) or lateral tail vein from rats under anesthesia. Whole blood (target volume of 1 mL) samples were collected into tubes with no anticoagulant, allowed to clot for 30 min at room temperature, and centrifuged (1750× *g*) for 15 min at 2 °C–8 °C to yield serum. Each serum sample was divided into 2–3 aliquots, frozen on dry ice and then stored at −80 °C until testing.

Terminal sampling of whole blood (target minimum of 6 mL) was collected from the cranial vena cava or other suitable peripheral vessel and processed as above. Before each blood collection, the animals were anesthetized using isoflurane (target 5%) via inhalation. The animals were sacrificed using an overdose of sodium pentobarbital after terminal blood collection.

### 5.6. Body Weights

Individual body weights were recorded for all animals during the pre-test period for group assignment and dose-volume calculation.

### 5.7. Serum Concentration for Pharmacokinetic Analysis

Serum samples were analyzed for each individual monoclonal antibody (mAb) using ECL immunoassays on the Meso Scale Discovery (MSD, Gaithersburg, MD, USA) platform. Individual assays were used to measure the concentration of antibody in rat serum. Each antibody was measured using individual domains that bound only one antibody in the drug product. Biotinylated and ruthenylated domains were used directly. Standards and Quality Control samples (QCs) were prepared by spiking known amounts of antibody combination drug into rat serum. The stock calibration standard, quality control samples (QCs), and study samples were diluted in assay dilution buffer (ADB) at the minimum required dilution. The initial diluted stock calibration standard was further serially diluted using Sample Dilution Buffer (SDB) (ADB containing 5% (*v/v*), 2% (*v/v*), or 10% (*v/v*) rat serum-depending on the assay) to generate a calibration curve. Samples that required higher dilution were diluted using SDB before analysis.

For the XA-a assay, the domain was directly coated to an MSD plate, and then blocked with Blocking Buffer (phospho buffered saline (PBS) containing 3% *w/v* bovine serum albumin). For the XA-b assay, biotin-domain conjugate was added to blocked MSD streptavidin plates. The standards, QCs and samples were diluted, added to the plates and incubated on a plate shaker at room temperature. After incubation, for the XA-a and XA-b assays, ruthenium conjugated secondary anti-human antibody was added, incubated and shielded from light. 

For the XCD-a, XCD-c, and XCD-d assays, biotinylated domain was added to streptavidin plates. After incubation and blocking, standards, QCs, study samples and ruthenylated domain were added and incubated shielded from light. 

For the XA-c, XB, XE and XCD-b assays, a solution of reaction mixture (biotinylated and ruthenylated domains plus standards, QCs or study samples) was incubated at room temperature on a plate shaker and shielded from light. After the initial incubation, the reaction mixture was loaded to a blocked, MSD streptavidin-coated plate and incubated at room temperature on a shaker shielded from light. 

For all assays, after incubations and the addition of proprietary MSD Read Buffer, a chemiluminescent signal was generated when an electric current was applied and was detected for all wells using an MSD Sector Imager Instrument. The resulting signal was measured in ECL units, and the concentrations of antibodies were interpolated from the calibration curve.

### 5.8. Immunogenicity

Serum samples were evaluated for the presence of rat anti-human anti-drug antibodies (ADA) to each of the antibodies for each animal using an ECL assay. For the assay, a mixture of biotinylated or ruthenium conjugated antibodies was prepared in ADB and added to each well of a 96-well polypropylene plate. Diluted samples were transferred to the plates containing the labeled antibodies and incubated on a plate shaker. MSD streptavidin-coated plates were blocked with a blocking buffer. Following incubation, the blocked streptavidin-coated plates were washed three times in Wash Buffer (PBS containing 0.05% *v/v* Tween-20). The incubated mixtures were transferred to the washed blocked streptavidin plates and incubated at room temperature on a plate shaker shielded from the light. The streptavidin plates were washed three times in Wash Buffer. After washing, MSD Read Buffer was added to each well. ECL signals were determined for all wells using an MSD Sector Imager Instrument. The assay cut point was calculated for each plate by adjusting the normal control serum from each plate with a correction factor. Samples with an ECL signal greater than the assay cut-point were reported as positive.

Screened positive samples were further tested in the competitive confirmation assay with and without antibody. All assay procedures were the same as described above except the sample preparation procedure. Quality control and study samples were initially diluted 1:5 in ADB. To generate the 1:10 diluted samples without drugs, an aliquot of each the 1:5 diluted samples was mixed with an equal volume of ADB. To generate the 1:10 diluted samples with drugs, a separate aliquot of the same 1:5 diluted samples was mixed with equal volume of ADB containing the drug mAbs for a final dilution of 1:10 with the drug. The diluted samples were then incubated at 37 °C for 1 h. All subsequent sample incubation and sample analysis on MSD Sector Imager Instrument procedures were the same as in the screening assay. Percent Inhibition (%INH) was calculated for all samples using the formula, %INH = [1 − (sample with drug/sample without drug)] × 100%. Samples with %INH greater than the confirmation cut point were reported as positive for the presence of anti-mAb antibodies and further analyzed using the titer assay described below. Samples with %INH at or below the confirmation cut point were reported as negative.

All ADA-positive samples from all assays were further analyzed for titer determination using the ECL assay described above. Study samples were diluted 1:10 using ADB to generate the first dilution of the titration curve. Diluted samples were then diluted using SDB. The reported end-point titer was determined by performing linear regression using the two points flanking the signal intercept. The plate-specific cut-point was used as the signal intercept.

### 5.9. Pharmacokinetic Analysis

Non-compartmental PK analyses were performed on the serum concentration-time data to calculate T_1/2_, C_max_, area under the serum concentration curve (AUC), the apparent volume of distribution (V), and clearance (Cl). All serum concentrations that were less than the LLOQ after the first measurable concentration were excluded from further calculations. Terminal elimination half-life values were determined from the slope of the line in the terminal elimination phase of the serum concentration-time curve. The T_1/2_ values and other terminal phase parameters were not reported if the r-value of the best-fit line was <0.8. A minimum of three time-points in the terminal phase were required for the calculation of T_1/2_.

## Figures and Tables

**Table 1:** Pharmacokinetic parameters of NTM-1631 for ADA-negative animals.

Antibody	Dose mg/kg	Sex	N	T_1/2_ day	C_max_/Dose kg × µg /mL/mg	AUC∞/Dose Day × kg × µg /mL/mg	CL mL/day/kg	Vc mL/kg
XA-a	0.1	M	14	17.23	8.957	85.6955	3.85	95.75
	0.1	F	15	15.56	11.4517	104.6601	3.15	70.79
	1	M	7	16.97	10.00825	63.3717	4.15	101.62
	1	F	9	15.26	10.79059	97.5364	3.16	69.58
	10	M	5	15.26	9.568051	62.5584	4.86	107.07
	10	F	10	14.61	10.454225	87.4375	3.37	71.04
	Mean			15.82	10.18	83.5433	3.76	85.98
	SE			0.43	0.36	7.09	0.27	7.09
XA-b	0.1	M	14	15.82	8.445	76.6225	4.33	98.88
	0.1	F	15	14.66	11.1411	94.1734	3.5	74.11
	1	M	7	14.74	9.18426	60.5433	5.03	106.89
	1	F	9	13.4	9.92303	84.5505	3.86	74.56
	10	M	5	14.45	16.9725	57.6311	5.97	124.38
	10	F	10	13.78	16.8571	68.8351	4.5	89.5
	Mean			14.48	12.0872	73.7260	4.53	94.72
	SE			0.34	1.57	5.77	0.36	5.88
XA-c	0.1	M	14	16.17	8.87910	60.6509	5.39	125.66
	0.1	F	15	15.33	12.73490	73.7408	4.48	98.95
	1	M	7	14.39	8.71959	50.8748	6.02	124.88
	1	F	9	12.36	11.69086	71.4821	4.69	83.64
	10	M	5	14.52	8.23345	44.0113	6.84	143.27
	10	F	10	13.68	9.17088	54.0151	5.88	116.08
	Mean			14.41	9.90480	59.13	5.55	115.41
	SE			0.54	0.69	4.38	0.33	7.91

Vc = Volume of distribution of the Central Compartment, CL = Clearance, C_max_ = maximum observed mean concentration, AUC∞ = Area under the curve from time = 0 to infinity.

**Table 2:** Pharmacokinetic parameters of NTM-1632 for ADA-negative animals.

Antibody	Dose mg/kg	Sex	T_1/2_ Day	C_max_/Dose kg × µg /mL/mg	AUC∞/Dose Day × kg × µg /mL/mg	CL mL/kg/Day	Vc mL/kg
XB-a	0.1	M	17.9	11.1	77.8	4.29	33.5
	0.1	F	17.5	12.4	86	3.88	30.5
	1	M	16	9.83	77.6	4.3	35.3
	1	F	15.1	9.92	62.4	5.35	37.5
	10	M	14.7	9.865	72.8	4.59	36.4
	10	F	13.6	8.82	68.79	4.85	41.2
	Mean		15.9	10.32	74.23	4.54	35.73
	SE		1	1.25	8.18	0.51	3.63
XB-b	0.1	M	9.51	11	51.5	6.48	33.7
	0.1	F	20.3	12.3	58.1	5.75	30.6
	1	M	13.3	10.4	53.8	6.2	35.9
	1	F	14.2	9.71	42.1	7.94	38
	10	M	11.7	10.035	49.49	6.75	36.6
	10	F	11.4	9.013	47.76	6.98	40.1
	Mean		13.40	10.41	50.46	6.68	35.82
	SE		3.75	1.14	5.45	0.75	3.33
XB-c	0.1	M	19.6	10.5	79.3	4.21	34.9
	0.1	F	18.7	12.6	91.5	3.65	29.9
	1	M	16.8	9.35	80.4	4.15	37.3
	1	F	16.7	9.59	65.4	5.11	39.1
	10	M	13.7	8.995	79.16	4.22	40
	10	F	13.3	8.427	76.62	4.35	43.1
	Mean		16.47	9.91	78.73	4.28	37.38
	SE		2.56	1.49	8.35	0.47	4.57

C_max_ = Maximum observed mean concentration; AUC∞ = Area Under the Curve to infinity; CL = Clearance; Vc = Volume of distribution of the central compartment.

**Table 3:** Pharmacokinetic parameters of NTM-1633 for ADA-negative animals.

Antibody	Dose mg/kg	T_1/2_ day	C_max_/Dose kg × µg /mL/mg	AUC∞/Dose Day × kg × µg /mL/mg	CL mL/kg/day	Vc mL/kg
XE-a	0.1	21.7	21.6	81.8	4.09	22.8
	1	13.9	9.5	75.2	4.43	38.2
	10	16.3	8.02	80.9	4.12	44.3
	Mean	17.3	13.04	79.3	4.21	35.1
	SE	2.3	4.3	2.1	0.11	6.4
XE-b	0.1	30.1	8.71	40.9	8.18	43.4
	1	10.7	10.8	45.9	7.26	32.7
	10	11.8	8.18	45.7	7.29	38.1
	Mean	17.5	9.23	44.17	7.58	38.1
	SE	6.3	0.8	1.63	0.3	3.1
XE-c	0.1	8.19	6.15	18.7	17.9	54.4
	1	22.5	7.52	20.8	16	47.1
	10	9.91	6.28	17.2	19.4	54.8
	Mean	13.5	6.65	18.9	17.8	52.1
	SE	4.5	0.44	1.04	1	2.5

C_max_ = Maximum observed mean concentration; AUC∞ = Area Under the Curve to infinity; CL = clearance; Vc = volume of distribution of the central compartment; T_1/2_ = half-life.

**Table 4:** Pharmacokinetic parameters of NTM-1634 for ADA-negative animals.

Antibody	Dose mg/kg	T_1/2_ day	C_max_/Dose µg × kg/mL/mg	AUC∞/Dose Day × kg × µg /mL/mg	CL mL/day/kg	Vc mL/kg
	0.1	5.19	6.75	19.2	53.4	373
XCD-a	1	7.58	6.96	25.5	39.2	429
	10	7.47	6.16	25.4	39.4	425
Mean		6.747	6.62	23.37	44.00	409.00
SE		1.349	0.41	3.61	8.14	31.24
	0.1	7.57	6.10	29.6	34.6	370
XCD-b	1	11.6	6.77	49.7	20.2	338
	10	11.1	6.56	49.0	20.4	328
Mean		10.090	6.48	42.77	25.07	345.33
SE		2.197	0.34	11.41	8.26	21.94
	0.1	10.8	6.52	32.1	32.7	461
XCD-c	1	10.1	7.34	47.5	21.1	307
	10	10.2	5.71	44.5	22.5	331
Mean		10.367	6.52	41.37	25.43	366.33
SE		0.379	0.82	8.16	6.33	82.86
	0.1	7.3	6.55	24.5	45.8	386
XCD-d	1	9.6	7.42	41.7	24.0	335
	10	9.9	6.31	42.2	23.7	338
mean		8.933	6.76	36.13	31.17	353
SE		1.422	0.58	10.08	12.67	28.62

C_max_ = Maximum observed mean concentration; AUC∞ = Area Under the Curve to infinity; CL = clearance; Vc = volume of distribution of the central compartment; T_1/2_ = half-life.

## Supplementary Materials

The following are available online at https://www.mdpi.com/2072-6651/11/6/345/s1, Table S1: Numbers of ADA-positive animals.
