# Supplementary material for: Pharmacokinetics of Human Recombinant Anti-Botulinum Toxin Antibodies in Rats
Source: Toxins (Basel). 2019 Jun 17;11(6):345. doi: 10.3390/toxins11060345 (PMC6628388; doi:10.3390/toxins11060345)
Supplement: Supplementary file 1 [file toxins-11-00345-s001.pdf]

Supplementary Materials: Pharmacokinetics of Human Recombinant Anti-Botulinum Toxin Antibodies in Rats

Yero Espinoza, David Wong, Ago Ahene, Kenneth Der, Zachary Martinez, John Pham, Ronald R. Cobb, Shauna Farr-Jones, James. D. Marks and Milan T. Tomic

Table S1. Numbers of ADA-positive Animals.

Dos	Sex	Antibody Titer per Time-point (Study Day)					
		**Pre**	**29**	**36**	**43**	**57**	**72**
NTM-1631							
	Males	0/3	0/3	0/3	0/3	1/3	0/3
0.1 mg/kg	Females	0/3	0/3	0/3	0/3	0/3	0/3
	Total	0/6	0/6	0/6	0/6	1/6	0/6
	Males	0/3	2/3	2/3	2/3	0/3	2/3
1 mg/kg	Females	0/3	1/3	1/3	2/3	1/3	1/3
	Total	0/6	3/6	3/6	4/6	1/6	3/6
	Males	0/3	2/3	3/3	1/3	3/3	1/3
10 mg/kg	Females	0/3	0/3	1/3	1/3	2/3	1/3
	Total	0/6	2/6	4/6	2/6	5/6	2/6
NTM-1632		Pre	29	36	43	57	72
	Males	0/3	0/3	0/3	0/3	1/3	0/3
0.1 mg/kg	Females	0/3	0/3	0/3	0/3	0/3	0/3
	Total	0/6	0/6	0/6	0/6	1/6	0/6
	Males	0/3	2/3	2/3	2/3	0/3	2/3
1 mg/kg	Females	0/3	1/3	1/3	2/3	1/3	1/3
	Total	0/6	3/6	3/6	4/6	1/6	3/6
	Males	0/3	2/3	3/3	1/3	3/3	1/3
10 mg/kg	Females	0/3	0/3	1/3	1/3	2/3	1/3
	Total	0/6	2/6	4/6	2/6	5/6	2/6
NTM-1633		Pre	29	36	43	57	72
	Males	0/3	0/3	0/3	0/3	0/3	0/3
0.1 mg/kg	Females	0/3	0/3	0/3	0/3	0/3	0/3
	Total	0/6	0/6	0/6	0/6	0/6	0/6
	Males	0/3	2/3	2/3	2/3	0/3	2/3
1 mg/kg	Females	0/3	1/3	1/3	2/3	1/3	1/3
	Total	0/6	3/6	3/6	4/6	1/6	3/6
	Males	0/3	2/3	3/3	1/3	3/3	1/3
10 mg/kg	Females	0/3	0/3	1/3	1/3	2/3	1/3
	Total	0/6	2/6	4/6	2/6	5/6	2/6
NTM-1634		Pre	29	36	43	57	72
	Males	0/3	0/3	0/3	0/3	1/3	0/3
0.1 mg/kg	Females	0/3	0/3	0/3	0/3	0/3	0/3
	Total	0/6	0/6	0/6	0/6	1/6	0/6
	Males	0/3	2/3	2/3	2/3	0/3	2/3
1 mg/kg	Females	0/3	1/3	1/3	2/3	1/3	1/3
	Total	0/6	3/6	3/6	4/6	1/6	3/6
	Males	0/3	2/3	3/3	1/3	3/3	1/3
10 mg/kg	Females	0/3	0/3	1/3	1/3	2/3	1/3
	Total	0/6	2/6	4/6	2/6	5/6	2/6

[1]With one exception, the serum samples are from different animals at each timepoint. Serum samples taken at pre-doses and on Day 29 are from the same animals.
